# Oligonucleotide array discovery of polymorphisms in cultivated tomato (*Solanum lycopersicum *L.) reveals patterns of SNP variation associated with breeding

**DOI:** 10.1186/1471-2164-10-466

**Published:** 2009-10-09

**Authors:** Sung-Chur Sim, Matthew D Robbins, Charles Chilcott, Tong Zhu, David M Francis

**Affiliations:** 1 Department of Horticulture and Crop Science, The Ohio State University, Ohio Agricultural Research and Development Center, 1680 Madison Ave, Wooster, OH 44691, USA; 2 Syngenta Biotechnology, Inc, 3054 East Cornwallis Road, Research Triangle Park, NC 27709, USA

## Abstract

**Background:**

Cultivated tomato (*Solanum lycopersicum *L.) has narrow genetic diversity that makes it difficult to identify polymorphisms between elite germplasm. We explored array-based single feature polymorphism (SFP) discovery as a high-throughput approach for marker development in cultivated tomato.

**Results:**

Three varieties, FL7600 (fresh-market), OH9242 (processing), and PI114490 (cherry) were used as a source of genomic DNA for hybridization to oligonucleotide arrays. Identification of SFPs was based on outlier detection using regression analysis of normalized hybridization data within a probe set for each gene. A subset of 189 putative SFPs was sequenced for validation. The rate of validation depended on the desired level of significance (α) used to define the confidence interval (CI), and ranged from 76% for polymorphisms identified at α ≤ 10^-6 ^to 60% for those identified at α ≤ 10^-2^. Validation percentage reached a plateau between α ≤ 10^-4 ^and α ≤ 10^-7^, but failure to identify known SFPs (Type II error) increased dramatically at α ≤ 10^-6^. Trough sequence validation, we identified 279 SNPs and 27 InDels in 111 loci. Sixty loci contained ≥ 2 SNPs per locus. We used a subset of validated SNPs for genetic diversity analysis of 92 tomato varieties and accessions. Pairwise estimation of θ (*F*st) suggested significant differentiation between collections of fresh-market, processing, vintage, Latin American (landrace), and *S. pimpinellifolium *accessions. The fresh-market and processing groups displayed high genetic diversity relative to vintage and landrace groups. Furthermore, the patterns of SNP variation indicated that domestication and early breeding practices have led to progressive genetic bottlenecks while modern breeding practices have reintroduced genetic variation into the crop from wild species. Finally, we examined the ratio of non-synonymous (Ka) to synonymous substitutions (Ks) for 20 loci with multiple SNPs (≥ 4 per locus). Six of 20 loci showed ratios of Ka/Ks ≥ 0.9.

**Conclusion:**

Array-based SFP discovery was an efficient method to identify a large number of molecular markers for genetics and breeding in elite tomato germplasm. Patterns of sequence variation across five major tomato groups provided insight into to the effect of human selection on genetic variation.

## Background

Tomato is an important vegetable crop contributing pro-vitamin A and vitamin C to the human diet and providing high economic value to producers. Tomato has also been extensively used as a model organism for basic studies in plant biology, with a focus on resistance to pests, plant development, and biochemical pathways. As a result, extensive genetic and genomic resources have been developed. In the early 1990's, a high-resolution genetic map was constructed using more than 1,000 RFLP markers between *Solanum lycopersicum *and a wild relative, *S. pennellii *[1]. The first plant resistance (R) gene to be isolated and cloned, *Pto *conferring resistance to the bacterium *Pseudomonas syringae *pv. *tomato*, was characterized via map-based cloning in tomato [2]. To date, several other R-genes from tomato have been cloned including genes conferring resistance to fungal (*Cf-9*, *Cf-2*, and *Ve1*), insect (*Mi*), and viral (*Sw5 *and *Tm2*^2^) pathogens [3-8]. Genes regulating growth habit (*sp*) and fruit development (*fw2.2*, *ovate*, and *sun*) have also been cloned and characterized [9-12]. Genome sequencing projects are adding new resources for genetic analysis. Recently, large-scale sequencing of tomato ESTs identified 609 potential simple sequence repeats (SSRs) and 152 PCR-based polymorphic markers that were mapped on the *S. lycopersicum *× *S. pennellii *reference population [13].

During and following domestication, tomato has undergone intensive selection and cultivated varieties have narrow genetic diversity relative to other crops. This narrow diversity makes it difficult to identify molecular markers that are polymorphic in modern breeding material. For instance, of the 609 putative SSRs that were identified based on bioinformatic screening of EST databases, only 61 are polymorphic in cultivated tomato [13] and only 10 to 25 of these SSRs are polymorphic within a given cross (Francis, unpublished). The low level of polymorphism has resulted in a limited application of marker-assisted selection (MAS) in populations derived from elite by elite crosses due to a scarcity of markers. In order to identify enough markers for genetic mapping and MAS, genome wide approaches to screening for markers must be adopted.

Single nucleotide polymorphisms (SNPs) are the most common type of sequence variation and tend to be biallelic in plant species [14]. New methods for SNP detection are facilitating high-throughput genotyping, and provide strong motivation for the identification of sequence variation. In tomato, an *in silico *approach for SNP discovery was employed utilizing publicly available EST sequences [15]. This study identified 1,245 contigs with three EST sequences from each of two *S. lycopersicum *varieties, Rio Grande and TA496. One SNP was detected for every 8,500 bp analyzed, with 101 candidate SNPs in 44 genes. This strategy was limited by the predominance of TA496 sequences in the EST databases at the time. A second strategy to facilitate SNP discovery was developed based on conserved orthologous set (COS) introns [16]. A total of 1,487 SNPs were detected in 302 loci among 12 tomato varieties (3 fresh-market, 6 processing, 1 vintage, 1 *S. lycopersicum *var *cerasiformae*, and 1 *S. pimpinellifolium*). Of these, 579 SNPs in 162 loci were polymorphic within elite material. SNPs were detected in the COS introns at a rate 5.3 fold higher than in coding regions. These studies suggest that sufficient polymorphism exists in tomato to expand MAS to elite by elite crosses, but the primary limitation is the development of efficient methods to identify large numbers of SNPs.

A high-throughput approach based on an oligonucleotide array was proposed to identify sequence polymorphism including SNPs and insertion/deletions (InDels) in haploid yeast [17]. Borevitz et al [18] referred to polymorphisms discovered from array hybridizations as single feature polymorphisms (SFPs) and identified nearly 4,000 potential SFPs between two *Arabidopsis *varieties, Columbia (Col) and Landsberg *erecta *(Ler). Array-based SFP detection has been applied to several plant species including barley [19-21], rice [22], and cowpea [23].

In the present study, we report SFP discovery using oligonucleotide arrays hybridized with genomic DNAs from three *S. lycopersicum *varieties representing fresh-market, processing, and cherry (*S. lycopersicum *var. *cerasiformae*) for marker development that will benefit both geneticists and breeders. We verified 114 SFPs in 111 loci and conducted a genetic diversity analysis using 92 tomato varieties and accessions with 51 loci discovered from the arrays.

## Results

### Prediction and validation of SFPs

We called putative SFPs from two-way comparisons between the fresh-market variety FL7600 and the processing genotype OH9242 (FO), between the cherry accession PI114490 and FL7600 (PF), and between PI114490 and OH9242 (PO) using a regression based method at six levels of significance from α = 10^-2 ^to α = 10^-7^. A total of 210 SFPs were selected for sequence validation. High quality sequence data covering the target SFP probe regions were obtained for 189 of these, while partial sequences that did not extend into the target regions were obtained for an additional 21 loci. Validation rates were determined based on the 189 SFPs (Table 1). The validation rates for six CI levels were 60% (α ≤ 10^-2^), 63% (α ≤ 10^-3^), 71% (α ≤ 10^-4^), 74% (α ≤ 10^-5^), 76% (α ≤ 10^-6^), and 75% (α ≤ 10^-7^). Validation rate reached a plateau between α ≤ 10^-4 ^and α ≤ 10^-5 ^(Table 1). Although validation rates increased, at higher CI there was a reduction in the rate of known SFPs called (i.e. an increase in Type II error). At α ≤ 10^-3^, 11% of known polymorphism were excluded. This rate increased to 17% at α ≤ 10^-4^, 23% at α ≤ 10^-5^, 32% at α ≤ 10^-6^, and 47% at α ≤ 10^-7^. The increase in Type II error leads to a wide range in the estimate of the number of SFPs for the six CI levels from 344 (α ≤ 10^-7^) to 5111 (α ≤ 10^-2^) (Table 1).

**Table 1:** SFP detection among three varieties of cultivated tomato and sequence validation

**Stringency of confidence interval**	**Validation rate^1^**	**No. of SFPs**			**Total^2^**
			
		**FL7600 vs. OH9242**	**FL7600 vs. PI114490**	**OH9242 vs. PI114490**	
α ≤ 10^-2^	60%	3467 (2080)^3^	3725 (2235)	3585 (2151)	8518 (5111)
α ≤ 10^-3^	63%	1023 (644)	1493 (941)	1336 (842)	2970 (1871)
α ≤ 10^-4^	71%	435 (309)	823 (584)	736 (523)	1481 (1052)
α ≤ 10^-5^	74%	217 (161)	523 (387)	490 (363)	885 (655)
α ≤ 10^-6^	76%	135 (103)	402 (306)	336 (255)	629 (478)
α ≤ 10^-7^	75%	95 (71)	284 (213)	263 (197)	458 (344)

^1^Validation rate was calculated based on the sequence data for 189 putative SFPs

^2^Number of SFPs across all three two-way comparisons without redundancy

^3 ^Number in parenthesis indicates the number of SFPs corrected by the corresponding validation rate

### SFP based discovery of SNPs and InDels

**Figure 1:**
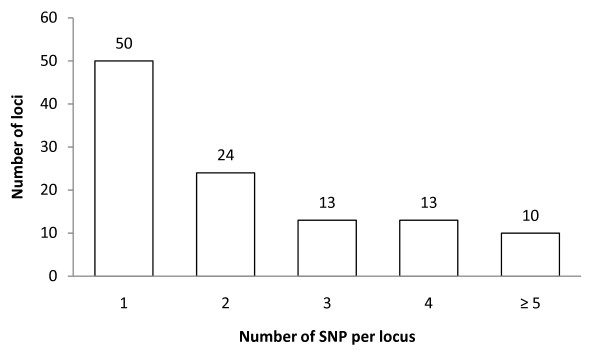
**Frequency of the number of SNPs detected per locus among three tomato varieties, FL7600, OH9242, and PI114490**.

**Figure 2:**
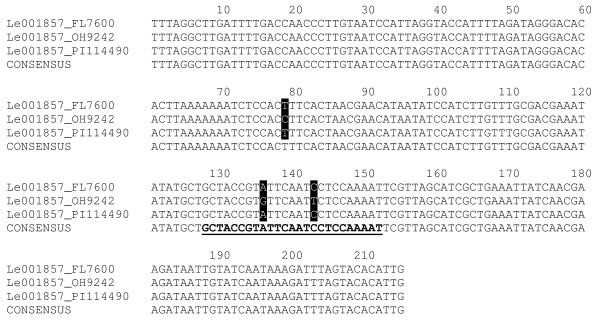
**Alignment of amplicon sequences derived from FL7600, OH9242, and PI114490 from probe number 5 of the Le001857 locus**. SNPs among the three varieties are highlighted in black. The probe position is bold and underlined.

A total of 108 SNPs and five InDels were detected in the target probe regions. An additional 171 SNPs and 22 InDels were identified in sequences flanking probes identified as SFPs. Thus, a total of 279 SNPs and 27 InDels were identified in 111 genes, 110 of which contained at least one SNP and one of which contained only InDels (see Additional files 1 and 2). These sequence polymorphisms were not evenly distributed among loci as multiple SNPs (≥ 2 per locus) were identified in 60 loci including 24 loci with 2 SNPs, 13 loci with 3 SNPs, 13 loci with 4 SNPs, and 10 loci with ≥ 5 SNPs (Figure 1). An example of a single locus, Le001857, containing multiple SNPs is shown in Figure 2. This locus (SGN unigene ID: SGN-U317952) encodes 1-aminocyclopropane-1-carboxylic acid synthase that is presumably involved in the ethylene biosynthetic process. The alignment of sequences from three varieties at this locus showed two SNPs at the target SFP probe position and an additional SNP located 5' of the probe. Two varieties, FL7600 and PI114490 share a common haplotype at this locus, while OH9242 has a second haplotype (Figure 2). Further analysis indicates that the second SNP (C/T) in the probe sequence is a non-synonymous substitution (Pro/Ser) while the other two SNPs (A/G and T/C) are synonymous substitutions.

### Genetic diversity in tomato germplasm

In order to evaluate the utility of SNPs discovered from array hybridization and subsequent sequence validation, we used 46 markers to genotype a collection of 92 tomato varieties and accessions representing different market uses and origins (23 fresh-market, 40 processing, 20 vintage, 5 landrace, and 4 *S. pimpinellifolium*) (see Additional file 3). We tested the hypothesis that these groups, based on origin and breading history, represent genetic subpopulations. To assess whether breeding practices have affected how variation is partitioned between the groups, we estimated pairwise θ (*F*st) according to Weir and Cockerham [24] using the MSA software package [25]. Pairwise estimates of θ ranged from 0.61 to 0.83 between *S. pimpinellifolium *and the four cultivated groups of *S. lycopersicum *(Table 2) indicating a high level of differentiation between cultivated tomato and this wild relative. The processing varieties represented a distinct subpopulation relative to fresh-market (θ = 0.23, *P *= 0.001), vintage (θ = 0.28, *P *= 0.001), and landrace (θ = 0.30, *P *= 0.001) entries included in the analysis. Fresh-market varieties represented a distinct subpopulation relative to vintage (θ = 0.30, *P *= 0.001) and landrace (θ = 0.27, *P *= 0.003) (Table 2). Expected heterozygosity, the probability of selecting two alleles at random from each subpopulation [26], was estimated across all 46 loci as 0.141 ± 0.028 (standard error) for fresh-market, 0.154 ± 0.027 for processing, 0.059 ± 0.019 for vintage, 0.133 ± 0.032 for landrace, and 0.319 ± 0.041 for *S*. *pimpinellifolium*.

**Table 2:** Pairwise estimates of θ (*F*st) among the five groups of 92 tomato varieties and accessions

	**Processing (40)^1^**	**Vintage (20)**	**Landrace (5)**	***S. pimpinellifolium *(4)**
Fresh-market (23)	0.23 (0.001)^2^	0.30 (0.001)	0.27 (0.003)	0.69 (0.002)
Processing		0.28 (0.001)	0.30 (0.001)	0.68 (0.001)
Vintage			0.26 (0.015)	0.83 (0.003)
Landrace				0.61 (n.s.)

^1^Number in parenthesis indicates the number of varieties and accessions of each group used in this study

^2^Number in parenthesis indicates *P*-value for 10,000 permutations with bonferoni correction. The *P*-values above 0.05 are presented as 'n.s.'

Allele-based analysis of the 46 marker loci and haplotype-based analysis using five additional loci detected variation (two alleles or haplotypes) within the fresh-market varieties for 25 of 51 loci (see Additional file 4). For 23 out of the 25 loci polymorphic in the fresh-market varieties, allelic variation was also present among the processing varieties. The two exceptions were Le003743 and Le005962. The most common fresh-market alleles were present in a lower frequency for Le000287, Le022048, Le001857, Le006551, Le003155, Le002822, Le000231, and Le006853 in the processing germplasm. Additional allelic variation (two alleles) was detected in the processing varieties at Le018657, Le012281, Le001470, Le002898, Le005879 and Le013946, but these loci were monomorphic in the fresh-market germplasm. Of the 31 loci that were polymorphic within elite germplasm, we detected only a single allele (A_1_) in the vintage and landrace varieties for 15 loci (see Additional file 4). In contrast, six loci (Le000343, Le010828, Le000076, Le012794, Le000721, and Le004122) contained allelic variation within the vintage or landrace varieties, but not in the fresh-market and processing varieties.

The 88 varieties of *S. lycopersicum *(excluding PI 114490) showed no allelic variation for 14 loci: Le003224, Le009197, Le016246, Le005826, Le000146, Le001991, Le006791, Le009598, Le000035s, Le000209, Le003096, Le004420, Le004777, and Le016258 (see Additional file 4). The four accessions of *S. pimpinellifolium*, however, showed allelic variation (two or three alleles) for these 14 loci and 16 other loci.

Of 110 loci with SNPs, 23 loci contained four or more polymorphisms. We included four additional loci that correspond to LEOH ESTs with > 4 SNPs [15] and, as a control, the Rcr3 gene, which is required for Cf-2 resistance and is thought to be under diversifying selection [27,28]. We considered these genes to be highly polymorphic and were interested in addressing whether they might contain an unusual number of changes that could modify protein function. We eliminated all loci with InDel polymorphisms, and the remaining set of 20 loci was used to estimate the ratio of non-synonymous (Ka) to synonymous substitutions (Ks). Estimates of the Ka/Ks ratio for the 20 loci ranged from 0 (Ka = 0 and Ks > 0) to > 1 (Ka > 0 and Ks = 0) (Table 3). The values for Le003313 (0.609) and Le003743 (0.636) are in the same range estimated for Rcr3 (0.692). The sequences for Le011957 (0.904), Le006895 (1.412), Le013904 (> 1), Le013946 (> 1), Le004790 (> 1), and Le007111 (> 1) had values that approach or exceed 1. These loci have a higher than expected number non-synonymous changes.

**Table 3:** Summary statistics of Ka and Ks for 20 loci with multiple SNPs

**Locus**	**SFP sequence ID**	**SGN unigene ID^1^**	**Chromosome**	**No. of SNPs**	**Ka**	**Ks**	**Ka/Ks**	**SGN annotation**
Le011123	Le011123	SGN-U598411	1	4 (3)^2^	0.009	0.016	0.579	dehydration-responsive related protein
Le006895	Le006895	SGN-U569640	2	4 (4)	0.009	0.006	1.412	cytochrome P450 76C2
Rcr3	Le013093	SGN-U331451	2	10 (4)	0.009	0.013	0.692	cysteine protease (Rcr3)
Le013887	Le013887	SGN-U600765	2	7 (4)	0.008	0.119	0.063	unknown
Le002348	Le002348	SGN-U588569	3	4 (4)	0.007	0.051	0.137	lipid transfer protein 6 (LTP6)
LEOH8	Le009961	SGN-U313561	3	4 (4)	0.002	0.018	0.111	plasma membrane intrinsic protein 2A (PIP2A)
Le013904	Le013904	SGN-U571948	3	4 (4)	0.016	0.000	> 1	unknown
Le001310	Le001310	SGN-U312733	4	4 (1)	0.010	0.049	0.203	ribosomal protein L17
Le006853	Le006853	SGN-U585308	4	5 (4)	0.007	0.055	0.121	oligopeptide transporter OPT protein
Le013946	Le013946	SGN-U342731	4	6 (6)	0.016	0.000	> 1	fertility restorer-like protein
LEOH38	Le003743	SGN-U314789	5	5 (5)	0.007	0.011	0.636	ripening regulated protein (DDTFR6/A)
Le004790	Le004790	SGN-U566816	5	4 (3)	0.015	0.000	> 1	ubiquinol-cytochrome C chaperone protein
Le007111	Le007111	SGN-U577041	5	4 (4)	0.060	0.000	> 1	unknown
Le009852	Le009852	SGN-U577041	5	8 (2)	0.000	0.059	0.000	unknown
LEOH35	Le003313	SGN-U318485	9	8 (8)	0.014	0.023	0.609	photosystem II reaction centre W (PsbW)
LEOH31	Le004579	SGN-U317091	9	10 (9)	0.007	0.057	0.123	putative chlorophyll synthetase
Le000343	Le000343	SGN-U578047	unknown	4 (4)	0.000	0.000	0.000	metallothionein-like protein type 2
Le005230	Le005230	SGN-U585097	unknown	4 (3)	0.009	0.033	0.280	nuclear transport factor 2 (NTF2)
Le006861	Le006861	SGN-U578820	unknown	18 (4)	0.004	0.014	0.286	phosphatidylinositol-4-phosphate 5-kinase
Le011957	Le011957	SGN-U589331	unknown	4 (4)	0.015	0.016	0.904	phosphoinositide-specific phospholipase C

^1^SOL Genomics Network (SGN:) unigene ID

^2^Number in parenthesis indicates the number of SNPs in an open reading frame (OFR) of each locus

## Discussion

Array-based SFP discovery proved to be a high-throughput approach to develop new molecular markers for genetics and breeding in tomato. We used genomic DNA as a hybridization target to detect SFPs on an Affymetrix (Santa Clara, CA) array. Validation rate leveled off between 71% (α ≤ 10^-4^) and 75% (α ≤ 10^-7^) for predicted SFPs between three cultivated tomato varieties. However, using α values between 10^-6 ^and 10^-7 ^resulted in a high percentage of known SFPs within probe features being excluded.

Our empirically determined estimate of the efficiency of random sequencing in cultivated tomato is 3.5% SNP discovery on a per gene (EST) basis, an estimate that is influenced by the 1/8,500 bp occurrence of polymorphism and the unequal distribution of polymorphism within genes [15]. A random sequencing approach is therefore highly inefficient for SNP discovery within cultivated tomato. The efficiency of random sequencing would increase to 18%-19% on a per gene basis if non-coding sequences were targeted [16]. The use of array hybridization to improve the rate of SFP validation to over 70% represents a dramatic improvement in efficiency relative to random sequencing.

The complexity of the target, the method of detection (algorithm), and stringency of probability impact validation rate. Complexity of the target is dependent on whether cDNA (mRNA) or genomic DNA is used and on genome size when genomic DNA is the target. Using mRNA as a hybridization target has been used to reduce the complexity of large plant genomes. This approach, however, adds other issues due to presence of multi-gene families, variation in the level of expression, and post-transcriptional sequence polymorphism [21,23]. The robustified projection pursuit (RPP) method has been developed as a way to improve SFP detection with fewer biological replicates [19]. Projection pursuit analyses also perform well under a range of distributions [29], a feature that is of particular importance to hybridizations using mRNA target where the range of expression must be considered. Using RPP and selecting probes with overall outlying scores (u) from the 5% distribution tail, the validation rate in Barley was 80% [19]. Using the same method for Cowpea, but selecting u from the 15% distribution resulted in a validation rate of 67% [23]. Thus, the stringency of selection is a key feature of increasing reducing false discovery rate. SFP detection accuracy for which known SNP genotypes are predicted from mRNA hybridizations are reported to be as high as 95% when multiple methods are used [20]. These values drop to ~80% when a single method is used [20]. Our detection of SFPs using DNA as the target was less sensitive than similar studies in *A. thaliana *(97%) [18]. We attribute this difference to the complexity of the target, which is approximately 950 Mb for tomato and 125 Mb for *A. thaliana*. Our validation rate was comparable to the 75% found for rice, which has a genome size of 400 Mb [22]. Increasing the stringency (lowering the α value) decreased the false discovery rate while increasing Type II error, an observation that is expected. Values of α between 10^-4 ^and 10^-5 ^provided the best balance between false discovery and eliminating true polymorphisms.

The SNPs discovered from array hybridization provided a tool to both estimate θ (*F*st) and inspect allele distribution within and between groups in order to assess the affects of selection during the breeding history of cultivated tomato. Selection of individuals with favorable mutations during domestication and through breeding practices has led to a reduction of genetic diversity in crop species [30]. A narrow genetic base has previously been reported in cultivated tomato [31,32]. It is postulated that genetic bottlenecks occurred during domestication and during the introduction of tomato to Europe from Latin America by Spanish explorers [31,33]. The patterns of lower SNP variation we observed in vintage and landrace groups relative to wild tomatoes document a genetic bottleneck. However, breeding practices have stressed the introgression of new genetic variation, especially for disease resistance from wild species [34,35]. Tomato breeding for fresh-market and processing varieties diverged with a strong emphasis on distinct ideotypes reinforced by the initiation of mechanical harvest. Efforts to develop tomatoes specifically for mechanical harvest were initiated in 1943, but did not produce acceptable varieties until the mid 1960s [36]. Given the historical practices of tomato breeding that include introgression and market differentiation, we hypothesize that genetic differentiation may have occurred between varietal classifications and that elite germplasm may contain more variation relative to landrace and vintage varieties. Our pairwise estimates of θ between the five subpopulations representing fresh-market, processing, vintage, landrace, and *S. pimpinellifolium *strongly suggest genetic differentiation has occurred due to breeding. Furthermore within subpopulation estimates of genetic diversity provide evidence that modern breeding practices have broadened the genetic diversity of tomato relative to landrace and vintage varieties. These results are consistent with previous findings [31,32,37].

We also investigated whether a subset of highly polymorphic (≥ 4 SNPs) genes might contain functional changes. We identified six loci with high ratios of non-synonymous substitution relative to our control gene. Proteins encoded by these genes include, a phosphoinositide-specific phospholipase C (Le011957), a cytochrome P450 (Le006895), a fertility restorer-like protein (Le013946), a ubiquinol-cytochrome C chaperone protein (Le004790), and two proteins of unknown function (Le013904 and Le007111). These genes may be candidates for functional analysis in order to identify genes that contribute to existing phenotypic variation in crop plants.

Plant genomes have evolved under human selection. Perhaps the best-documented consequence of this selection is a reduction of variation caused by genetic bottlenecks during the domestication process and through selective sweeps due to linkage to genes that are desirable in agriculture [38]. Much of what we currently know about the genes that were selected during domestication and breeding derive from the map-based cloning of individual genes. Selection has often been toward loss-of-function mutations. Examples include the loss of seed dispersal in grains through shattering [39] and loss of branching [38]. At the same time, some desirable phenotypes are due to gain of function mutations. Examples include disease resistance [2], high beta-carotene content in tomato which is conferred by a promoter mutation leading to increased expression of the fruit-specific beta-cyclase [40], and elongated fruit shape due to the duplication, translocation, and subsequent over-expression of an IQ67 domain-containing gene in tomato fruit [12].

The idea that algorithms might be applied for the high throughput identification of the genes selected during crop improvement has been proposed for a number of plant species. The application of such approaches is somewhat crop specific, and is influenced by mating system and rates of polymorphism. In highly diverse species, such as maize, the focus has been to identify selective sweeps [38]. However in crops that have experienced severe genetic bottlenecks it will be difficult to distinguish selective sweeps from the effects of genetic drift due to the bottlenecks themselves. On the other hand, species with reduced genetic variation might offer a model to detect genes with increased levels of polymorphism. Arguably, these are the genes that are of most interest to plant breeders as they likely contribute to existing phenotypic variation. In the case of tomato, an obstacle will be to distinguish genes that are associated with introgressions due to linkage disequilibrium from the selected genes themselves.

## Conclusion

We demonstrated that SFP discovery using an oligonucleotide array is an efficient way to develop a large number of markers that may be used for high-resolution genetic mapping and marker-assisted breeding in elite tomato germplasm. The SNPs and InDels detected in this study can be a useful resource for haplotyping and population genetic studies. We combined the identification of genetic variation within genes with methods to investigate the effects of selection on cultivated tomato. These methods included genetic diversity analysis to detect genes with unequal distribution between five subpopulations; and finally, an analysis of substitution rates was applied to genes with multiple polymorphisms to identify genes in which sequence variation may have functional consequences. We conclude that breeding has increased genetic diversity in modern tomatoes relative to vintage tomato varieties that were selected prior to the widespread application of Mendelian principles to breeding [41]. Our analysis identified some alleles in vintage varieties that have apparently been lost in modern tomatoes. However such reductions in genetic variation are offset by an overall increase in allelic diversity in both fresh-market and processing varieties. Based on the frequent presence of these "new" alleles and haplotypes in wild relatives of cultivated tomato, we conclude that increased allelic diversity is most likely due to the purposeful introgression from wild species.

## Methods

### Plant material

Germplasm used in this study included 88 varieties of *S. lycopersicum *and four accessions of *S. pimpinellifolium *(see Additional file 3). The *S. lycopersicum *germplasm consisted of 23 fresh-market, 40 processing, 20 vintage, and five landrace varieties. Fresh-market and processing varieties were selected from public breeding efforts that release commercially relevant parents and hybrids. Several processing lines were donated directly by seed companies. In addition, selected inbred lines were obtained through single-seed descent and sequential self-pollination of commercial hybrids.

### Affymetrix oligonucleotide array

The custom designed Affymetrix array contained 22,821 probe sets of 11 perfect match and 11 mismatched probes each. The probe sets correspond to 22,714 unigenes assembled based on ESTs available in 2002 from mixed genetic backgrounds (predominately TA496). Genomic DNA from the three *S. lycopersicum *varieties, FL7600 (fresh-market), OH9242 (processing), and PI114490 (var. *cerasiformae*) was labeled according to a modified version of the BioPrime DNA Labeling System (Invitrogen, Carlsbad, CA) protocol. The genomic DNA was denatured in the presence of random octamers and fragmented. Briefly, 4 μl of DNA (500 ng/μl) was mixed with 20 μl of 2.5× random primer solution and 20 μl nuclease free water in a 44 μl reaction at 4°C. The contents were mixed by vortexing. The reaction was carried out using a thermocycler programmed for 99°C for five minutes. Biotin labeled dNTP mixture (5 μl) and Klenow Fragment (1 μl) were added to the 44 μl sample on ice. The contents were mixed by vortexing. The reaction was carried out in a thermocycler for two hours at 37°C. A portion of the labeling reaction was run on an agarose gel to verify the expected band size of approximately 150 to 300 bp. Each of the three samples was hybridized to the custom Syngenta Tomato Genome Array (Affymetrix, Santa Clara, CA) in triplicate for 16 hours at 45°C with 60 rpm in an Affymetrix Hybridization Oven 640. The washing and staining of the arrays with streptavidin-phycoerythin (SAPE) was conducted in an Affymetrix Fluidics Station 450, according to the manufacturer's recommendations. The processed arrays were scanned using Affymetrix GeneChip^® ^Scanner 3000.

### Single feature polymorphism (SFP) prediction

The hybridization data for perfect match (PM) probes was extracted from the raw MicroStation Cell Library (CEL) files containing data for FL7600, OH9242, and PI114490. Three files representing three independent hybridizations were available for each variety. Following extraction, data were background corrected and quantile normalized using the Bioconductor package implemented in the R language [42]. These data were then analyzed using statistical models implemented in the SAS software package (SAS Institute, Inc., Cary, NC) to predict SFPs. The PM data from three replicates of each variety were first transformed using log_10 _(L_10_PM). The coefficient of variance (CV) for each probe was also calculated by dividing the standard deviation by the mean of the L_10_PM data based on three replicates. Probes with CV > 0.1 were eliminated from further consideration. The L_10_PM data were standardized to a mean of zero and a standard deviation of 1 (sPM), and data were analyzed to predict single feature polymorphisms (SFPs). We used a regression-based approach to identify potential SFPs. This approach was based on the identification of probes within a given gene set that fall outside of a confidence interval (CI) at six defined levels of significance ranging from α = 10^-2 ^to α = 10^-7^. The confidence interval (CI) for a value of X was determined by the relationship a + b*X - t(α/2) * √ [(1 + h) * MSE] and the upper level of the confidence interval a + b*X + t(α/2) * √ [(1 + h) * MSE], with the value of the t statistic changed to generate a CI at the desired level of significance (α). Because we defined both upper and lower confidence limits, the t statistic was adjusted for the one-tailed test by choosing t for (α/2).

### SFP validation and SNP discovery

Primers flanking target regions of 210 randomly-selected potential SFPs were designed using BatchPrimer3 v1.0 software [43] with the optimal PCR product length between 150 and 400 bp. PCR reactions were conducted in a total volume of 50 μl containing 10 mM Tris-HCl (pH 9.0 at room temperature), 50 mM KCl, 1.5 mM MgCl_2_, 50 μM of each dNTP, 0.1 μM of each forward and reverse primers, 20 ng of DNA template, and 1 unit of *Taq *DNA polymerase. Amplification was performed in a thermocycler (MJ Research, Inc., Watertown, MA) programmed for 3 min at 94°C followed by 40 cycles of 45 s at 94°C, 45 s at a suitable annealing temperature between 56 and 60°C, and 1 min 45 s at 72°C, followed by an extended incubation for 6 min at 72°C. PCR products were purified using QIAquick PCR purification columns and QIAEX II Gel Extraction kits (Qiagen, Inc., Valencia, CA) or simply by ethanol precipitation. Purified PCR products were sequenced using the Big-Dye Termination cycle sequencing reactions and an ABI Prism 3100xl sequencer (Applied Biosystems, Inc., Foster city, CA). Sequencing was performed in the forward and reverse directions for each of the PCR products for two or three varieties of tomato. The Pregap4 module of the Staden sequence analysis package [44] was used to align sequences to determine if the potential SFPs contained sequence polymorphisms. Probe positions were overlaid with sequence data in order to determine whether SFP outliers were validated. All sequence data has been submitted to GenBank GSS database (FI855394 to FI855581).

### Analysis of genetic diversity in a tomato germplasm collection

Genetic differentiation was assessed between the five groups, fresh-market, processing, vintage, landrace, and *S. pimpinellifolium*, based on pairwise θ (*F*st) according to Weir and Cockerham [24]. The 92 varieties and accessions were genotyped using 43 SNP and three InDel markers identified as SFPs in the analysis of the array hybridization data. Genotyping was performed using one of three platforms. For SNPs that were easily and cost-effectively scored as cut amplified polymorphisms (CAPs), amplicons were restriction digested and separated on agarose gels. Insertion/deletion polymorphisms (InDels) were detected using a LI-COR IR2 model 4200 fragment analysis system (LI-COR Biosciences, Lincoln, NE). Finally, SNPs that could not be screened as CAPs were genotyped using a single-base extension assay implemented using the LUMINEX 200 (Luminex, Corp., Austin, TX). For the analysis described here, we chose genes that overlapped with previously developed markers based on analysis of the EST database [15,45] or mRNA hybridizations to a Nimblegen expression array (manuscript in preparation). Expected heterozygosity was used to infer genetic diversity within each group. The estimates of pairwise θ (*F*st) and heterozygosity were obtained using Microsatellite analyzer (MSA) V4.05 [25]. The *P*-value for the pairwise θ was based on 10,000 permutations and a bonferoni correction. Expected heterozygosity was calculated based on a single locus for each group and values for 46 markers were then averaged to estimate heterozygosity across all markers.

We investigated the distribution of haplotypes in the tomato collection directly by sequencing or indirectly by SNP genotyping. Loci Le006861 and Le016258 were investigated by direct sequencing for the 31 varieties and accessions. As we identified only two haplotypes within cultivated tomatoes, subsequent haplotyping was investigated through SNP genotyping. The loci Le011957, Le013904, and Le013946 each contain ≥ 4 SNPs. Two SNP markers were developed for each of the three loci and SNP detection for the 82 varieties and accessions was performed with the Luminex 200 system as described above.

### Estimation of the ratio of non-synonymous to synonymous substitutions

In order to identify coding sequences for loci with ≥ 4 SNPs, we used Basic Local Alignment Searches (BLAST) against the tomato EST database. Alignments were used to identify and remove intron sequences, and open reading frames within the exon sequence were subsequently identified using ORF finder . The two sequences that contained both alleles at each SNP were trimmed so that no stop codons were present. DNA sequences were aligned using ClustalX v1.8 [46], and the alignment files were saved as the input files for estimation of synonymous (Ks) and non-synonymous (Ka) substitution rates using K-estimator 6.0 [47,48].

## Authors' contributions

SCS, extracted data and performed background correction and normalization, improved statistical models, identified putative SFPs, performed sequence validation, and drafted the manuscript. MDR, assisted in the analysis of sequence data and manuscript preparation. CC and TZ performed microarray hybridizations. DMF, developed statistical models, designed and supervised the research.

All authors have read and approved the final manuscript.

## Supplementary Material

Additional file 1 **SNP variation for three tomato varieties, FL7600, OH9242, and PI114490 used in SFP discovery**. The data includes locus names and SNP positions, flanking sequences, and primers. Click here for file

Additional file 2 **InDel variation for three tomato varieties, FL7600, OH9242, and PI114490 used in SFP discovery**. The data includes locus names and InDel positions, flanking sequences, and primers. Click here for file

Additional file 3 **Description and SNP genotypes of plant materials used in this study**. The data includes categorical details of 92 tomato varieties and accessions with genotypes at 46 loci. Click here for file

Additional file 4 **Summary of allelic diversity analysis at 51 loci**. The summary shows allelic diversity of 92 tomato varieties and accessions divided into five groups, fresh-market, processing, vintage, landrace, and wild species. Click here for file
